# Pandemic Grief Scale in detection of grief reaction among physicians in COVID-19 era

**DOI:** 10.1186/s43045-021-00130-8

**Published:** 2021-08-03

**Authors:** Samir El Sayed, Sarah Gomaa, Shereen Aboelfotoh, Mohamed El Wasify

**Affiliations:** 1 Department of Psychiatry, Faculty of Medicine, Mansoura University, Mansoura, Egypt; 2 M.D Psychiatry, Mansoura University Students’ Hospital, Mansoura University, Mansoura, Egypt

**Keywords:** COVID-19, Grief, Pandemic

## Abstract

**Background:**

Physicians are considered one of the most vulnerable groups who might develop pandemic grief during this critical time of COVID-19 infection, and this grief reaction might have deleterious effects on their life. This cross-sectional observational online study aimed to investigate the pandemic grief reaction among physicians and its burden on their aspects of life.

**Results:**

Socio-demographic characteristics of 900 physicians were collected. The Pandemic Grief Scale (PGS) was used to detect the grief reaction among them and also Patient Health Depression Questionnaire-9 (PHQ-9) was used to evaluate the depressive manifestations. Sheehan Disability Scale was considered to investigate the burden of this grief on different aspects of life. The study revealed high mean score of Pandemic Grief Scale: 11.12 ± 2.34; the mean score of Sheehan Disability Scale was 17.63 ± 5.17, and the mean score of Patient Health Depression Questionnaire-9 was 19.89 ± 4.27.

**Conclusion:**

Pandemic grief is commonly experienced by the physicians during this COVID-19 era due to sudden loss of loved one or the cases themselves. This pandemic grief has drastic effect on domains of physicians’ life.

## Background

According to WHO, there are 161,513,458 confirmed cases of COVID-19 including 3,352,109 death cases [1]. People responded variably to the death of a loved one. Many experienced distorted functioning while others experienced initial dysfunctioning decreasing in the later months after this loss, also approximately 10% of bereaved individuals were overwhelmed by chronic and devastating distress [2].

Recent research showed that acute grief reaction after COVID-19 mortality was worse than death a result of other natural causes [3]. Socio-functional impairment is a diagnostic criterion in psychiatric disorders including impairments in social, occupational, and other domains of functioning [4].

Updated study and case reports indicated that functional impairment is a crucial compound of the consequences of the grief after COVID-19 death [5].

A sudden loss, in intensive care unit with other psychosocial stressors like catching infection, lockdown, and economic burden have been considered triggering factors in severe grief reactions in COVID-19 bereavement [6].

In a study conducted by Lai and colleagues composed of 1257 healthcare workers reached to the results that general distress was estimated in 72% of the study group, followed by depressive manifestations (50%), anxious symptoms (45%), and difficulty in falling asleep (34%) (Lai J et al. 2019).

To our knowledge, there are no enough studies investigating the occurrence of grief reaction among physicians during COVID-19 pandemic.

We aimed in this study to investigate the COVID-pandemic grief reaction among physicians, its effects on development of psychiatric manifestations and implications on different domains of life in this important society group.

## Methods

### Study design

This is cross-sectional online study conducted via Google Document domain starting from 2nd of January 2021 to 13th of February 2021

### Study population

A convenient sample of 900 participants performed this online google document.

### Inclusion and exclusion criteria

Participants of sexes, age range from 25 to 65 years and all medical specialities were included.

Those have major psychiatric disorders, other general medical conditions like chronic diseases or under the effect of psychotropic medications were excluded from the study.

#### Socio-demographic characteristics

The study socio-demographic data included age, sex, marital status, residence, smoking, relation to the deceased, work position, duration of the grief, seeking psychiatric help, and COVID-19 status.

#### Pandemic Grief Scale (PGS)

A 5-item Likert rating scale using 4-point time-anchored scale that spans a 2-week period (0 = not at all to 3 = nearly every day), participants rated how frequently they experienced each grief symptom (Sherman A. Lee and Robert A. Niemeyer, 2020) [7].

#### Patient Health Depression Questionaire-9 (PHQ-9)

A 9-item Liker scale using 4-point time anchored scale assessing the depressive manifestations over the last 2 weeks (0 = not at all to 3 = nearly every day). Total score as the following:

A- 1–4 means minimal depression
B- 5–9 means mild depression
C- 10–14 means moderate depression
D- 15–19 means moderately severe depression
E- 20–27 means severe depression

Also, there are 10 questions of the scale which did not include in the total score but assess to what extent these manifestations affect the different domains of life [8].

#### Sheehan Disability Scale (SDS)

It is a self-report scale, in which the participant rates the extent to which work/school, social life, and home life of family responsibilities are impaired by his or her symptoms on a 10-point visual analog scale. This 10-point visual analog scale uses spatiovisual, numeric, and verbal anchors simultaneously to assess disability. The numeric ratings of 0–10 can be translated into percentage. The 3 items can be presented as a single dimensional measure of global functional impairment ranging from 0 (unimpaired) to 30 (highly impaired).

There is no cut-off point but researcher must pay attention to participant score 5 on any of the 3 domains because high scores associated with drastic functional impairment [9].

### Ethical consideration

(1) Local ethical committee approval was taken to conduct this study.

(2) Informed consent was obtained electronically from all participants after giving full data about the aim of the study.

(3) Patients were confirmed about the confidentiality of the data collected and that they were able to withdraw from the study at any time without any reasons.

(4)STROBE statement and Guidelines: **N/A**

### Statistical analysis and data interpretation

Data were fed to the computer and analyzed using IBM SPSS Corp. Released 2013. IBM SPSS Statistics for Windows, Version 22.0. Armonk, NY: IBM Corp. Qualitative data were described using number and percent. Quantitative data were described using mean and standard deviation for parametric data after testing normality using Kolmogrov-Smirnov test. Significance of the obtained results was judged at the (0.05) level.

### Data analysis

#### Qualitative data

- Chi-square test for comparison of 2 or more groups
- Monte Carlo test as correction for chi-square test when more than 25% of cells have count less than 5 in tables (> 2*2).

### Quantitative data between groups

#### Parametric tests

- Student’s *t* test was used to compare 2 independent groups

##### Correlation

Pearson’s correlation

The Pearson product-moment correlation is used to determine the strength and direction of a linear relationship between two normally distributed continuous variables.

## Results

Table 1 showed the socio-demographic data of the studied group in which the mean age was 42.96 ± 10.62; the study sample composed of 504 males (56.0%) and 396 females (44.0%). Seven hundred sixty-five (85.0%) of the sample lived in urban areas while 135 (15.0%) lived in rural areas. One hundred twenty-one (13.4%) were single, 722 (80.2%) were married, 40(4.4%) were divorced, and 17 (1.9%) were widowed.

**Table 1:** Socio-demographic characteristics of the studied sample (*N* = 900)

Age/years:		
mean ± SD	42.96 ± 10.62	
(range)	(25–65)	
Sex:		
Male	504	56.0
Female	396	44.0
Residence:		
Urban	765	85.0
Rural	135	15.0
Marital status:		
Single	121	13.4
Married	722	80.2
Divorced	40	4.4
Widow	17	1.9
Smoking:		
Non-smoker	657	73.0
Smoker	243	27.0
Relation to deceased:		
Immediate family member		
Extended family member		
Close friend	231	25.7
Acquaintance		
Others	365	40.6
	109	12.1
	93	10.3
	102	11.3
Position:		
Resident	54	6.0
Demonstrator	44	4.9
Assistant lecturer	55	6.1
Lecturer	121	13.4
Assistant prof	120	13.3
Prof	241	26.8
Specialist	80	8.9
Consultant	185	20.6
Pandemic Grief Scale: mean ± SD (range)	11.12 ± 2.34 (4.0–15.0)	
Duration of the grief:		
< 1	391	43.4
1–3	267	29.7
4–6	242	26.9
Seeking psychiatric help:		
No		43.3
Yes	390	56.7
	510	
Sheehan Disability Scale: mean ± SD (range)	17.63 ± 5.17 (6.0–26.0)	
Patient Health Questionnaire-9: mean ± SD (range)	19.89 ± 4.27 (8.0–27.0)	
Degree of impact on quality of life:		
Not difficult at all		
Somewhat difficult	267	29.7
Very difficult	170	18.9
Extremely difficult	239	26.6
	224	24.9
COVID-19 status:		
Negative	506	56.2
Positive	394	43.8

Regarding smoking status, 657(73%) were non-smoker while 243 (27%) were smokers. As regards the relation to the deceased, 231 (25.7%) were immediate family member, 365 (40.6%) were extended family members, 109 (12.1%) were close friends, 93 (10.3%) were acquaintances and 102 (11.3%) were others.

Regarding the work position, 54 (6%) were residents, 44 (4.9%) were demonstrator, 55 (6.1%) were assistant lecturers, 121 (13.4%) were lecturers, 120 (13.3%) were assistant professors, 241 (26.8%) were professors, 80 (8.9%) were specialists, and 185 (20.6%) were consultants.

### Duration of the grief reaction

The number of the participants < 1 month were 391 (43.4%), from 1 to 3 months were 267 (29.7%), and from 4 to 6 months were 242 (26.9%). For seeking professional psychiatric help, 510 (56.7%) sought professional help while 390 (43.3%) did not .

Regarding COVID-19 status, 394 (43.8%) were positive while 506 (56.2%) were negative.

The mean score of Pandemic Grief Scale was 11.12 ± 2.34 (mean ± SD), the mean score of Sheehan Disability Scale was 17.63 ± 5.17 (mean ± SD), and the mean score of Patient Health Depression Questionnaire-9 was 19.89 ± 4.27 (mean ± SD). Regarding the degree of impact on quality of life, 267 (29.75%) are not difficult at all, 170 (18.9%) somewhat difficult, 239 (26.6%) very difficult, and 224 (24.9%) extremely difficult.

Table 2 illustrated a statistically significant positive association between the position of the Associated Prof. and being tested positive with COVID-19. Also, there is a statistically significant positive association between the mean score of Pandemic Grief Scale and positive cases of physicians with COVID-19.

**Table 2:** Association between socio-demographic, Sheehan Disability Scale, Pandemic Grief Scale, and COVID-19 infection among studied group

Variables	COVID-19		Test of significance
	Negative *n* = 506	Positive *n* = 394	
Age/years	42.91 ± 10.58	43.02 ± 10.66	*t* = 0.155 *p* = 0.877
Sex			
Male	284(56.1)	220(55.8)	*χ*^2^ = 0.008
Female	222(43.9)	174(44.2)	*p* = 0.931
Residence			
Urban	425(84.0)	340(86.3)	*χ*^*2*^ = 0.921
Rural	81(16.0)	54(13.7)	*p* = 0.337
Marital status			
Single	67(13.2)	54(13.7)	*χ*^2^ = 0.084, *P* = 0.77
Married	404(79.8)	318(80.7)	*χ*^2^ = 0.11,*P* = 0.745
Divorced	22(4.3)	18(4.6)	*χ*^2^ = 0.03, *P* = 0.87
Widow	13(2.6)	4(1.0)	*χ*^2^ = 2.89, *P* = 0.09
Smokers			
Non-smoker	365(72.1)	292(74.1)	χ^2^ = 0.439
Smoker	141(27.9)	102(25.9)	*p* = 0.507
Relation to disease			
Immediate family member	119(23.5)	112(28.4)	*χ*^2^ = 2.79, *P* = 0.09
Extended family member	211(41.7)	154(39.1)	*χ*^2^ = 0.627, *P* = 0.428
Close friend	68(13.4)	41(10.4)	*χ*^2^ = , 1.91, *P* = 0.17
Acquaintance	55(10.9)	38(9.6)	*χ*^2^ = 0.358, *P* = 0.549
Others	53(10.5)	49(12.4)	*χ*^2^ = 0.848, *P* = 0.356
Position			
Resident	32(6.3)	22(5.6)	*χ*^2^ = 0.215, *P* = 0.642
Demonstrator	25(4.9)	19(4.8)	*χ*^2^ = 0.007, *P* = 0.935
Assistant lecturer	28(5.5)	27(6.9)	*χ*^2^ = 0.672, *P* = 0.412
Lecturer	71(14.0)	50(12.7)	*χ*^2^ = 0.342, *P* = 0.558
Assistant prof	54(10.7)	66(16.8)	*χ*^2^ = 7.08, *P* = 0.008*
Prof	135(26.7)	106(26.9)	*χ*^2^ = 0.006, *P* = 0.94
Specialist	46(9.1)	34(8.6)	*χ*^2^ = 0.058, *P* = 0.809
Consultant	115(22.7)	70(17.8)	*χ*^2^ = 3.34, *P* = 0.07
Pandemic Grief Scale	10.56 ± 2.38	12.06 ± 2.01	*t* = 9.95
			*p* < .001*
Duration of the grief			
< 1	225(44.5)	167(42.4)	*χ*^2^ = 1.49
1–3	153(30.2)	113(28.7)	*P* = 0.47
4–6	128(25.3)	114(28.9)	
Seeking psychiatric help			
No	224(44.3)	166(42.1)	*χ*^2^ = 0.412
Yes	282(55.7)	228(57.9)	*P* = 0.521
Sheehan Disability Scale	17.28 ± 5.29	18.08 ± 4.98	*t* = 2.31 *p* = 0.02*
Patient Heath Questionnaire-9	19.54 ± 4.33	20.34 ± 4.16	*t* = 2.79 *p* = 0.005*
Degree of impact on quality of life	*n* = 506	*n* = 394	
Not difficult at all			
Somewhat difficult	163(32.2)	104(26.4)	*χ*^2^ = 3.59, *P* = 0.058
Very difficult	100(19.8)	70(17.8)	*χ*^2^ = 0.58, *P* = 0.447
Extremely difficult	131(25.9)	108(27.4)	χ^2^=0.263, *P* = 0.608
	112(22.1)	112(28.4)	χ^2^=4.69, *P* = 0.03*

*t* Student’s *t* test, Chi-square test, parameters described as mean ± SD or as number and percentage

Also, there is a statistically positive association between mean score of Sheehan Disability Scale and positive cases of COVID-19.

Patient Health Depression Questionnaire-9 has a statistically significant association with being positive with COVID-19, also positive cases of COVID-19 cases have a statistically significant association with extremely difficult degree of impairment of domains of life.

Table 3 highlighted the statistically significant positive association between mean score of Patient Depression Health Questionnaire-9 and positive cases of COVID-19 among the studied group.

**Table 3:** Factors affecting Sheehan Disability Scale, Pandemic Grief Scale, and Patient Health Depression Questionnaire-9 among studied group

	Pandemic grief scale	Test of significance	Sheehan Disability Scale	Test of significance	Patient depression questionnaire	Test of significance
Age/years	*r* = 0.0 *p* = 0.769		*r* = 0.037 *p* = 0.265		*r* = 0.042 *p* = 0.211	
Sex						
Male	11.18 ± 2.25	*t* = 0.439	17.74 ± 4.99	*t* = 0.739	20.07 ± 4.17	*t* = 1.39
Female	11.26 ± 2.46	*p* = 0.661	17.49 ± 5.39	*p* = 0.460	19.67 ± 4.39	*p* = 0.166
Residence						
Urban	11.17 ± 2.35	*t* = 1.45	17.69 ± 5.16	*t* = 0.798	19.98 ± 4.19	*t* = 1.42
Rural	11.49 ± 2.28	*p* = 0.147	17.30 ± 5.24	*p* = 0.425	19.41 ± 4.68	*p* = 0.157
Marital status						
Single	11.13 ± 2.26	*F* = 0.086	18.11 ± 4.88	*F* = 1.50	19.52 ± 4.31	*F* = 0.605
Married	11.23 ± 2.37	*P* = 0.968	17.59 ± 5.19	*P* = 0.212	19.97 ± 4.27	*P* = 0.612
Divorced	11.18 ± 2.06		18.08 ± 4.93		20.10 ± 3.96	
Widow	11.35 ± 2.45		15.41 ± 6.38		19.12 ± 4.97	
Smokers						
Non-smoker Smoker	11.23 ± 2.35 11.19 ± 2.33	*t* = 0.262 *p* = 0.793	17.65 ± 5.21 17.58 ± 5.08	*t* = 0.179 *p* = 0.858	19.86 ± 4.28 19.97 ± 4.24	*t* = 0.345 *p* = 0.730
Relation to disease						
Immediate family member	11.39 ± 2.31	*F* = 0.711	17.71 ± 5.25	*F* = 1.14	19.96 ± 4.47	*F* = 1.65
Extended family member	11.25 ± 2.41	*P* = 0.584	17.88 ± 4.95	*P* = 0.336	20.08 ± 4.11	*P* = 0.160
Close friend	10.99 ± 2.41		16.99 ± 5.37		19.45 ± 4.26	
Acquaintance	11.06 ± 2.29		17.94 ± 4.97		20.37 ± 3.97	
Others	11.12 ± 2.17		16.97 ± 5.67		19.11 ± 4.58	
Position						
Resident	11.29 ± 2.14	*F* = 0.679	17.02 ± 5.32	*F* = 0.630	19.28 ± 4.39	*F* = 0.844
Demonstrator	11.50 ± 2.17	*P* = 0.690	18.09 ± 4.84	*P* = 0.731	19.59 ± 4.27	*P* = 0.551
Assistant lecturer	11.18 ± 2.61		17.35 ± 5.28		19.62 ± 4.43	
Lecturer	11.17 ± 2.32		17.48 ± 5.09		19.98 ± 4.31	
Assistant prof	11.43 ± 2.35		17.25 ± 5.26		19.52 ± 4.37	
Prof	11.14 ± 2.37		18.11 ± 5.05		20.34 ± 4.08	
Specialist	10.81 ± 2.42		17.78 ± 5.32		19.51 ± 4.91	
Consultant	11.32 ± 2.32		17.44 ± 5.32		20.0 ± 4.06	
COVID-19						
−ve	10.57 ± 2.38	*t* = 9.95	17.28 ± 5.29	*t* = 2.31	19.55 ± 4.33	*t* = 2.79
+ve	12.06 ± 2.02	*p* < 0.001*	18.08 ± 4.98	*p* = 0.02*	20.34 ± 4.16	*p* = 0.005*

*F* One-way ANOVA test, *t* Student’s *t* test, chi-square test, Monte Carlo, parameters described as mean ± SD or as number and percentage

Table 4 showed the statistically significant association between the mean score of Pandemic Grief Scale and mean scores of Patient Health Questionaire-9, Sheehan Disability Scale, and degree of impact of domains of life.

**Table 4:** Correlation matrix between Pandemic Grief Scale, Patient Health Depression Scale, and Sheehan Disability Scale among studied sample

		Pandemic Grief Scale	Patient Health Questionnaire	Sheehan Disability Scale
Pandemic Grief Scale	r	1.000		
	p	.		
Patient Health Depression Scale	r	.140^**^	1.000	
	p	< 0.001	.	
Degree of impact on life domains	r	.145^**^	.888^**^	
	p	< 0.001	< 0.001	
Sheehan Disability Scale	r	.113^**^	.810^**^	1.000
	p	< 0.001	< 0.001	

## Discussion

To our knowledge, there are no enough studies examining the COVID-19 pandemic grief among the physicians, so the aim of the study is to use the Pandemic Grief Scale as an innovative tool to investigate the COVID-19 grief among the physicians and its impacts on psychological, social, and physical domains of their life during this pandemic era.

The results of this study were in parallel with the study conducted by Selman et al. [10] noted physicians were the front liners during COVID-19, had developed pandemic grief because of deaths of patients, colleagues, and their own loved members. Although many researches were conducted regarding COVID-19 grief of non-medical personnel, studying pandemic grief among physicians needed more attention.

The study has a result of mean score of PGS among studied physicians and was more than the cut-off score which is in accordance with result of the study by **[**8] who concluded that 56.6% of the sample scored above the cut score of ‡7 on the PGS for clinically dysfunctional pandemic grief.

The high mean score of PGS occurred among physicians before 6 months after grief which is in accordance with the study conducted by [11] who noted that most of the grief cases occurred during the 1st half of the year after loss of a loved one.

The study has a high mean score of PGS among physicians who were diagnosed positive with COVID-19; this result is in agreement with the result of a study conducted by ([12]) in which those who were diagnosed with COVID-19 have had higher PGS scores than those who were not diagnosed. This might be attributed by their relation to the deceased and their own physical and psychological manifestations related to COVID-19 infection.

The study has a positive association between mean score of PGS and functional impairment by mean score of Sheehan Disability Scale which in parallel with the study by [13] who found severe functional distress after COVID-19 grief either psychically, psychologically, or even socially.

The current study concluded that a remarkable percentage of physicians experienced pandemic grief beyond 1 month, which is in harmony with a study carried out by Robinson [14] who noted that physicians who witnessed facing the patients’death, helplessness, crying, impaired concentration, and anxiety manifestations, the pandemic grief often persisted more than 1 month and might need professional psychiatric management.

Also, the study found that the pandemic grief reaction was obvious when the deceased was one of the family members either immediate or extended; this is in accordance with the findings of the study by Wallace et al. [15] noting that the end-of-life events and the relationships of the health care workers to their loved ones were noticed as a crucial factor in the detection of the magnitude and consequences of pandemic grief.

This study revealed high mean scores of Patient Depression Health Questionnaire-9 which are in harmony with the study carried by Lu et al. [16] concluding that physicians who faced COVID-19 pandemic grief could have negative emotions like loss of loved one, guilty feeling, frustration, fear, and depressive manifestations.

Also, the study has a higher mean score of Sheehan Disability Scale which in accordance with the study conducted by Mayland et al. [17] highlighting that health care workers during COVID-19 pandemic were more liable to have deleterious effect on their domains of quality of life including physical, psychological, and social aspects as well as burnout syndrome.

Also, the study have results of high mean scores of Sheehan Disability Scale and Patient Depression Health Questionnaire-9 in contrary to the results of the study carried out by [18] who found that the total scores of depressive manifestations, pandemic COVID-19 grief, and functional disability were below clinically significant levels of impairment.

PGS in this study was more evident if the loss was one of the family member either immediate or extended; this finding is in line with the study done by [19] and another study conducted by [20], both concluding that people who were woman, younger age, recently bereaved, bereaved by the loss of a partner or child, and bereaved due to an unnatural loss had higher symptom profile in comparison to the other classes.

In conclusion, pandemic grief was commonly observed among the studied physicians’ group; also, the depressive manifestations and impacts on different aspects of physicians’ life were highly reported, which were positively associated to pandemic grief.

## Conclusions

In conclusion, pandemic grief reaction is common among the physicians during the COVID-19 era because of loss of loved one, death of COVID-19 cases; also, this pandemic grief affected their different domains of life, so they must seek for professional help to overcome this COVID-19 pandemic grief.

### Study limitations

This is an online cross-sectional study so we need more follow-up studies, and also we need clinical operational evaluation and to study if the speciality of the physician will have a role in pandemic grief ,all these limitations will need further studies.

## Data Availability

The datasets used and/or analyzed during the current study are available from the corresponding author on reasonable request.

## Abbreviations

WHO: World health organization
COVID-19: Coronavirus diseases-19
APA: American psychiatric association
PGS: Pandemic grief scale
SDS: Sheehan disability scale
PHQ-9: Patient health depression questionnaire-9
